# Strategies and structures towards implementing the Preventive Health Care Act in Baden-Württemberg – a quality report

**DOI:** 10.17886/RKI-GBE-2018-059

**Published:** 2018-05-16

**Authors:** Anne Würz

**Affiliations:** Ministry of Social Affairs and Integration Baden-Württemberg, Department of Health

To accompany and implement Germany’s Preventive Health Care Act, Baden-Württemberg has expanded the existing structures. These efforts were guided by the will to strengthen a settings approach in health promotion and prevention, further develop early detection screening offers by statutory health insurance companies and improve co-operation between industrial health promotion and occupational health and safety. To prevent double structures, implementing these objectives jointly with stakeholders will require a structured exchange of information.

The federal state framework agreement (LRV) anticipates involving the foundation Stiftung für gesundheitliche Prävention Baden-Württemberg [1]. Founded in 2009 on the initiative of the state of Baden-Württemberg, the foundation has focused on measures and projects with a settings approach in line with the Ottawa Charter for Health Promotion. It plays a pivotal role in the implementation and co-ordination of projects involving different statutory health insurance companies and carriers. Based on co-operation in the context of the Preventive Health Care Act, the aim is now to intensify cross-carrier co-operation. It is the objective of the foundation to focus its projects and measures on socially vulnerable groups. To accompany LRV implementation pursuant to section 20 f of Book 5 of the German Social Code (SGB V) of the Preventive Health Care Act, as mandated by section 8 of Baden-Württemberg’s health law [2] – a committee for health promotion and prevention was established and began working in April 2016. It was involved in the process of developing the LRV and provides the link to the communal level and communal health conferences.

Baden-Württemberg aims to establish a transparent procedure to implement projects across different carriers, as well as to promote professional supervision and the sharing of knowledge through the committee on the developments in Baden-Württemberg. Through this structure and the strategies it is tied to, Baden-Württemberg aims to ensure the highest quality of health promotion and prevention jointly with stakeholders and those involved in the LRV.
